# Isosorbide‐Based Optically Clear Adhesives With Ultrahigh Transparency and Rapid Strain Recovery for Flexible Displays

**DOI:** 10.1002/advs.77348

**Published:** 2026-08-30

**Authors:** Yoonji Choi, Geonwoo Lee, Yeonseo Kim, Dahyun Jin, Jinhoon Lee, Ki Sung Jung, Dong Woo Kim, Myung‐Jin Baek, Dong Woog Lee

**Affiliations:** ^1^ School of Energy and Chemical Engineering Ulsan National Institute of Science and Technology (UNIST) Ulsan Republic of Korea; ^2^ Youngwoo Co, Ltd (YOUNGWOO) Gunpo‐si Gyeonggi‐do Republic of Korea

**Keywords:** crosslinker, flexible display, optically clear adhesives, recoverability, transparency

## Abstract

Flexible and foldable displays require optically clear adhesives (OCAs) that combine ultrahigh optical transparency with mechanical resilience under repeated deformation. However, conventional acrylic‐based OCAs often fail to achieve both properties simultaneously. Herein, we synthesized a polyurethane diacrylate (PUDA) crosslinker incorporating biomass‐derived isosorbide and photostable 1,3‐bis(isocyanatomethyl)cyclohexane (H_6_XDI) for flexible display adhesives. The segmented polyurethane architecture forms a mechanically flexible yet robust network, enabling rapid strain recovery while maintaining ultrahigh optical transparency. Furthermore, the incorporation of an isosorbide‐based PUDA crosslinker introduces a biomass‐derived component and preserves excellent optical properties due to its intrinsically low birefringence. As a result, the PUDA crosslinked OCA exhibits exceptional optical transmittance (99.8%) and maintains high transparency (98.6%) even at 50% tensile strain, alongside low modulus, stable adhesion, and clean debonding behavior. These results highlight the potential of PUDA OCAs for high‐performance and energy‐efficient foldable display applications.

## Graphical abstract

Biomass‐derived isosorbide‐based PUDA crosslinker enables optically clear adhesives with ultrahigh transparency (99.8%), rapid strain recovery, and stable adhesion for flexible displays.

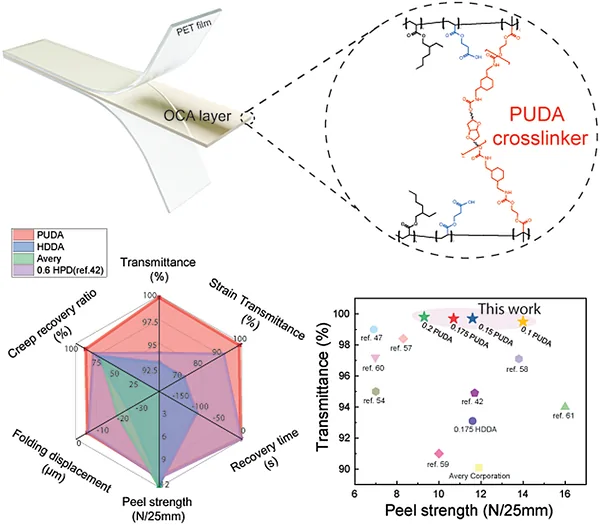

## 1 Introduction

Recent developments in flexible display technologies have increasingly focused on foldable and rollable form factors, which significantly enhance device portability and user convenience [1, 2]. However, these structural configurations simultaneously impose substantial mechanical stresses on the constituent materials. Therefore, materials employed in such devices must be capable of withstanding repeated mechanical deformation while maintaining stable mechanical integrity and optical performance [3]. These requirements are particularly critical for optically clear adhesives (OCAs), which are positioned directly along the light propagation path in multilayer display architectures [4]. All photons emitted from the organic light‐emitting diode (OLED) must pass through the adhesive layer before reaching the viewer [5]. Consequently, even small reductions in optical transmittance directly influence display luminance and overall energy consumption [6, 7, 8]. Because the adhesive layer is located between multiple functional layers and often near the top optical path of the display stack, maintaining ultrahigh optical transparency is a critical requirement [4].

At the device level, optical efficiency has a significant impact on power consumption and device performance [6]. Improving the optical transmittance of OCA can directly reduce OLED power consumption under identical luminance conditions [5]. Such improvements enhance visual clarity while lowering power demand, both of which are critical for reliable and energy‐efficient operation in flexible electronic devices [7]. Therefore, ultrahigh transparency adhesives are essential not only for improving display quality but also for enhancing the overall energy efficiency of flexible electronics.

Conventional acrylic‐based OCAs can provide adequate adhesion; however, maintaining high transparency under repeated bending remains a persistent challenge [9, 10]. As flexible display architectures evolve toward thinner and more highly integrated configurations, adhesive materials must preserve optical clarity while accommodating mechanical deformation [10]. In this context, urethane acrylate systems are widely employed in flexible and stretchable applications due to their tunable network architecture and mechanical compliance [11, 12]. Polyurethanes consist of soft polyol segments and rigid diisocyanate‐derived hard segments, and the microphase separation between these segments enables a balance of elasticity and mechanical strength [13]. Such a segmented structure is advantageous for flexible adhesive systems, as it allows the material to maintain structural stability while facilitating strain recovery under repeated deformation.

In addition, sustainability considerations are increasingly driving the incorporation of environmentally responsible components into advanced polymer systems [14, 15, 16, 17]. The incorporation of biomass‐derived components into polymer systems provides a pathway to reduce reliance on petroleum‐based resources while maintaining advanced functional properties [18]. Isosorbide, a rigid bicyclic diol derived from agricultural resources such as corn starch through glucose and sorbitol intermediates, has emerged as a promising bio‐based material [19]. Owing to its renewable origin, isosorbide has attracted considerable attention as a biomass‐derived building block for polymer systems [20]. Importantly, isosorbide has been reported to exhibit inherently low birefringence, which is advantageous for minimizing optical anisotropy and maintaining high optical transmittance in transparent materials [21, 22]. These characteristics make isosorbide particularly suitable for the design of ultrahigh‐transparency polyurethane networks [23]. The diisocyanate structure further influences optical durability and color stability [12]. Aliphatic diisocyanates are generally preferred over aromatic counterparts because of their resistance to yellowing and UV‐induced degradation [24]. Among them, 1,3‐bis(isocyanatomethyl)cyclohexane (H_6_XDI) offers a balanced combination of optical clarity and photochemical stability, while its rigid cycloaliphatic structure imparts excellent mechanical resilience, including rapid strain recovery and low hysteresis under repeated deformation, making it well suited for high‐transparency, flexible adhesive systems [25, 26, 27, 28].

Although polyurethane‐based OCA systems have been previously reported, these studies have primarily focused on recyclable on‐demand debonding [26], mechanical durability under impact [27], or processability [28], rather than simultaneously achieving ultrahigh optical transparency and rapid strain recovery required for repeated folding. Consequently, a polyurethane‐based OCA crosslinker capable of combining near‐perfect optical transmittance with rapid strain recovery under deformation, while incorporating bio‐based components, has yet to be demonstrated. Herein, we report a polyurethane diacrylate (PUDA) crosslinker incorporating biomass‐derived isosorbide and photostable H_6_XDI to enhance optical transparency of flexible display adhesives. The PUDA crosslinker forms a mechanically robust yet flexible network, enabling rapid strain recovery (< 2.5 s) while preserving ultrahigh optical transmittance under deformation. The resulting OCAs exhibit near‐perfect transparency, low modulus, stable adhesion, and clean debonding, highlighting their potential for energy‐efficient foldable display applications.

## 2 Results and Discussions

### 2.1 Characterization of Synthesized Crosslinker and OCAs

**FIGURE 1:**
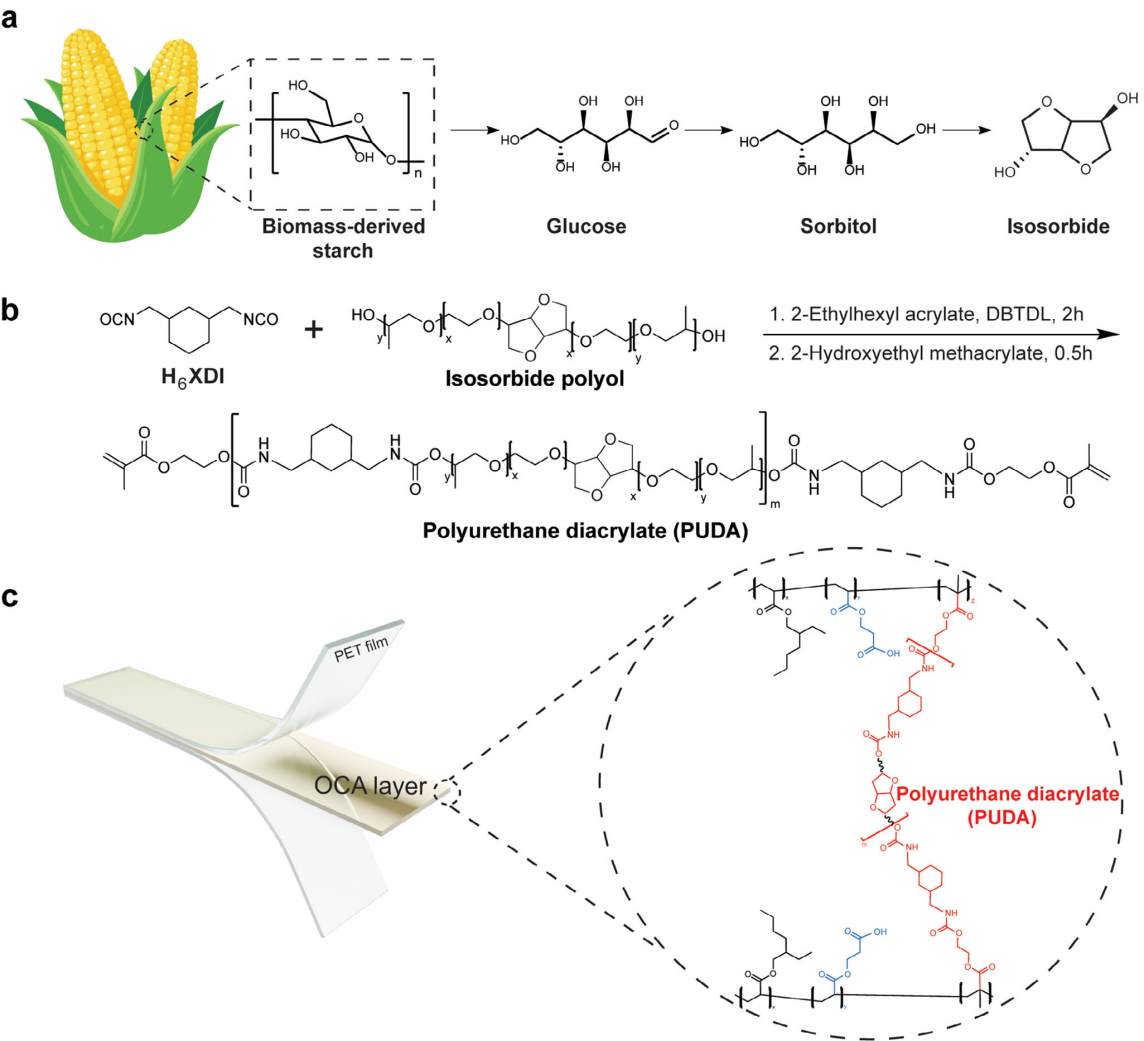
Schematic illustration of the bio‐based design, synthesis, and application of isosorbide‐based polyurethane diacrylate (PUDA) for optically clear adhesives (OCAs). (a) Biomass‐derived origin of isosorbide. (b) Synthetic route and molecular structure of PUDA. (c) Schematic illustration of a PUDA crosslinked OCA layer laminated with PET films.

As shown in Figure 1a, isosorbide is synthesized through a stepwise conversion process starting from glucose‐derived intermediates [23]. Subsequently, an isosorbide‐based polyol and H_6_XDI were used to prepare the PUDA crosslinker (Figure 1b). The PUDA was synthesized via a urethane reaction between the isosorbide polyol and H_6_XDI, followed by acrylation of the terminal hydroxyl groups to introduce acrylate functionalities. The corresponding PUDA crosslinked OCA structure is illustrated in Figure 1c. The chemical structure of the synthesized polyurethane diacrylate was confirmed by proton nuclear magnetic resonance (^1^H NMR). Vinyl proton peaks from the acrylate end groups appeared at 5.6 and 6.1 ppm with an approximately 1:1 integral ratio, indicating the successful introduction of acrylate functionalities (Figure S1). The progress of the urethane reaction was also tracked by FT‐IR spectroscopy, where the isocyanate (NCO) absorption band at 2264 cm^−^^1^ progressively decreased and eventually disappeared, confirming complete consumption of NCO groups during synthesis (Figure S2). The molecular weight of PUDA was determined by gel permeation chromatography, showing a molecular weight of approximately 44 000 g mol^−^^1^ (Figure S3 and Table S1).

OCA films were fabricated using 2‐carboxyethyl acrylate and 2‐ethylhexyl acrylate as monomers. As shown in Figure S4, the adhesion strength increases up to 40 mol% CEA and plateaus thereafter. Accordingly, a CEA:EHA ratio of 4:6 was selected, as it provides sufficient adhesion and good recoverability. The synthesized PUDA and conventional 1,6‐hexanediol diacrylate (HDDA) were used as crosslinkers (0.175 mol%) to examine the effect of crosslinker structure. The crosslinker compositions of the synthesized OCA formulations are summarized in Table 1.

**TABLE 1:** The crosslinker content of synthesized OCAs.

Crosslinker [mol%]	0.1 PUDA	0.15 PUDA	0.175 PUDA	0.2 PUDA	0.175 HDDA
**PUDA**	0.1	0.15	0.175	0.2	—
**HDDA**	—	—	—	—	0.175

To evaluate the crosslinking density, the gel fraction of each OCA was measured. As the PUDA content increased from 0.1 to 0.2 mol%, the gel fraction increased steadily from 67% to 86.3%, indicating a higher crosslinking density with increasing PUDA content. Specifically, gel fraction values of 67%, 80.3%, 84.9%, and 86.3% were obtained for 0.1, 0.15, 0.175, and 0.2 PUDA, respectively (Table S2).

For flexible display applications, OCAs must remain soft and deformable within the operating temperature range of −40°C to 85°C [29, 30]. The glass transition temperature (*T*_g_) of the OCAs was measured by differential scanning calorimetry (DSC) as a function of crosslinker type and content (Figure S5). The PUDA OCAs exhibited *T*_g_ values in the range of −57°C to −52°C, which are considerably lower than the *T*_g_ of HDDA OCA (−44.5°C). The lower *T*_g_ values observed for PUDA OCAs are attributed to the flexible polyurethane backbone and the much higher molecular weight of PUDA compared to HDDA, which can increase free volume and enhance polymer chain mobility [31]. In addition, a slight increase in *T*_g_ was observed with increasing PUDA content, which can be attributed to the increased crosslink density and the resulting restriction of chain mobility [32, 33]. Thermogravimetric analysis (TGA) was conducted to assess thermal stability (Figure S6). A minor weight loss was observed around 220°C due to the decomposition of the COOH groups in CEA [34, 35]. However, all PUDA OCAs showed their primary degradation event near 390°C, confirming that the OCAs possess sufficient thermal stability for practical display processing and operation.

The mechanical behavior of the OCAs was examined using a universal testing machine (UTM), and the resulting stress–strain curves are shown in Figure S7. Mechanical parameters including Young's modulus, ultimate tensile strength, elongation at break, and toughness are summarized in Table S3. As the PUDA content increased from 0.1 to 0.175 mol%, the OCAs exhibited progressively higher stress and reduced strain, indicating increased stiffness with increasing crosslink density. Accordingly, Young's modulus and ultimate tensile strength reached their maximum values at 0.175 mol% PUDA. When the PUDA content was further increased to 0.2 mol%, both stress and strain decreased compared to 0.175 mol%, accompanied by a reduction in Young's modulus and toughness. This trend is consistent with the rheological analysis shown in Figure S8. As presented in Figure S8a,c, both storage modulus (G′) and loss modulus (G″) increased with PUDA content up to 0.175 mol% and then decreased at 0.2 mol%. Overall, 0.175 PUDA provided the most balanced mechanical performance in terms of stiffness, strength, and extensibility.

### 2.2 Adhesive Properties of OCAs

**FIGURE 2:**
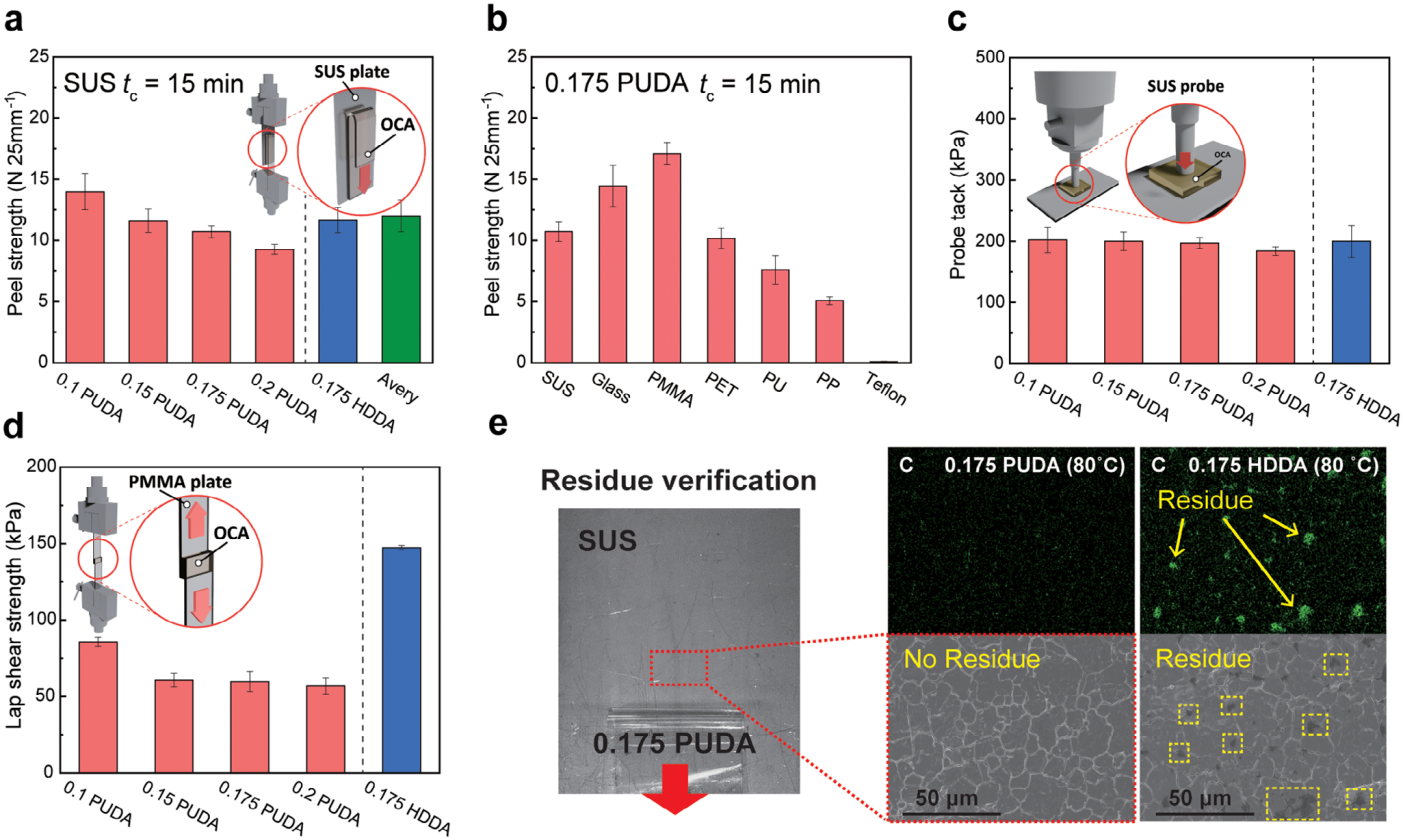
Adhesive performance of OCAs. (a) The peel strength of OCAs crosslinked with PUDA and HDDA, on stainless steel (SUS304). (b) The peel strength of 0.175 PUDA on different substrates. (c) The probe tack value and (d) the lap shear strength of OCAs crosslinked with PUDA and HDDA. (e) Scanning electron microscopy (SEM) images and energy‐dispersive X‐ray spectroscopy (EDS) mapping of stainless steel (SUS) surfaces after peeling of 0.175 PUDA and 0.175 HDDA at 80°C.

The adhesive performance of the OCAs was first evaluated by measuring the peel strength on stainless steel substrates as a function of crosslinker type and content, as shown in Figure 2a. The PUDA crosslinked OCAs exhibited a gradual decrease in peel strength with increasing PUDA content, showing values of 13.99 ± 1.48 N 25 mm^−1^ for 0.1 PUDA, 11.59 ± 0.95 N 25 mm^−1^ for 0.15 PUDA, 10.71 ± 0.50 N 25 mm^−1^ for 0.175 PUDA, and 9.28 ± 0.39 N 25 mm^−1^ for 0.2 PUDA (Table S4). The decrease in peel strength is attributed to the increased crosslinker content, which restricts polymer chain mobility and reduces interfacial wettability [36, 37, 38]. Despite this decrease, 0.175 PUDA maintained sufficient peel strength, exhibiting adhesion performance comparable to both the HDDA crosslinked OCA (11.65 ± 1.03 N 25 mm^−1^) and a commercial PSA from Avery Corporation (11.9 ± 1.30 N 25 mm^−1^). These results indicate that PUDA enables stable and practical adhesion performance under the investigated crosslinking conditions.

To further evaluate the applicability of the optimized formulation under representative temperature conditions relevant to flexible displays, temperature‐dependent 180° peel tests were conducted from −20°C to 60°C (Figure S9). Both the 0.175 PUDA and HDDA exhibited comparable peel adhesion at room temperature and 60°C, indicating stable adhesion performance under ambient and elevated temperature conditions. In contrast, a pronounced difference was observed under sub‐ambient conditions. The optimized 0.175 PUDA maintained a high peel strength of 45.8 N 25 mm^−^^1^ at −20°C, whereas the 0.175 HDDA exhibited a substantial reduction to 3.7 N 25 mm^−^^1^ under the same conditions. These findings suggest that the lower *T_g_* and more compliant polyurethane network of PUDA preserve sufficient segmental mobility and substrate conformability at low temperatures, thereby supporting its suitability for flexible display applications requiring robust low‐temperature performance.

To gain further insight into the origin of this temperature‐dependent adhesion behavior, oscillatory rheological measurements were performed under sub‐ambient (−5°C) and elevated temperature (40°C) conditions (Figure S10). Across the investigated temperature range, the 0.175 PUDA OCA consistently exhibited lower storage modulus (G′) values than the corresponding HDDA formulation. Notably, even under sub‐ambient conditions, the storage modulus of 0.175 PUDA remained substantially lower than that of 0.175 HDDA, indicating that the polyurethane network maintains a relatively compliant and deformable state at reduced temperatures. Furthermore, although both formulations exhibited increased storage moduli with decreasing temperature, the increase in G′ from room temperature to −5°C was considerably less pronounced for 0.175 PUDA, suggesting reduced temperature‐induced network rigidification and greater preservation of segmental mobility within the polyurethane network. These rheological observations are consistent with the peel adhesion results. Excessive rigidification of the HDDA network at low temperatures limits substrate wetting and adhesion, whereas the PUDA network maintains an appropriate balance between mechanical integrity and molecular mobility across the investigated temperature range.

Based on this balance between adhesion strength and network stability, the 0.175 PUDA formulation was selected for further evaluation on different substrates. Figure 2b and Table S5 summarize the peel strength of 0.175 PUDA measured on stainless steel, glass, poly(methyl methacrylate) (PMMA), poly(ethylene terephthalate) (PET), PU film, polypropylene (PP), and Teflon. 0.175 PUDA exhibited sufficient adhesion across all tested surfaces, demonstrating broad interfacial compatibility. Notably, PMMA showed the highest peel strength (17.11 ± 0.88 N 25 mm^−1^), followed by glass (14.45 ± 1.69 N 25 mm^−1^) and PET (10.18 ± 0.83 N 25 mm^−1^), likely due to enhanced interfacial bonding arising from polar interactions between the acrylate‐based adhesive and the ester functionalities of PMMA [39, 40]. In addition, the 0.175 PUDA exhibited reasonable adhesion strength to PU and PP, highlighting its suitability for plastic‐based flexible displays [9]. Although negligible peel strength was observed on Teflon, the overall results demonstrate that PUDA OCAs provide robust adhesion across a wide range of substrate chemistries.

The instantaneous adhesion behavior of the OCAs was evaluated by probe tack measurements, as shown in Figure 2c. Probe tack values are primarily governed by rapid interfacial wetting and the formation of physical interactions during short contact times [41]. The PUDA crosslinked OCAs exhibited probe tack values ranging from 184 to 202 kPa depending on PUDA content. (Table S4) The 0.1 PUDA sample showed the highest probe tack (202 ± 21 kPa), followed by a gradual decrease to 184 ± 7 kPa at 0.2 mol%. As predicted, this tendency indicates that increasing PUDA content slightly reduces segmental mobility at the interface, limiting instantaneous wetting while maintaining comparable initial adhesion performance. Overall, the probe tack values are sufficient to ensure adequate interfacial wetting and practical adhesion performance [42].

Lap shear tests were conducted to evaluate the shear‐dependent adhesion behavior of the OCAs. Lap shear strength is influenced by both the cohesive strength of the adhesive and the adhesive strength determined by the wettability and interfacial interactions (e.g., hydrogen bonding) with the substrate [43, 44, 45]. As shown in Figure 2d and Table S4, the 0.175 HDDA exhibited the highest lap shear strength (147.4 ± 1.4 kPa), whereas the PUDA OCAs showed lower values ranging from 85.7 ± 2.9 kPa at 0.1 mol% to 56.8 ± 5.2 kPa at 0.2 mol%. The higher lap shear strength of 0.175 HDDA can be attributed to its compact and densely packed network structure. Owing to its low molecular weight, 0.175 HDDA enables closer chain spacing within the network, which facilitates stronger intermolecular interactions such as hydrogen bonding and leads to higher cohesive strength, which is consistent with higher stiffness of 0.175 HDDA compared to that of 0.175 PUDA measured with UTM (Figure S7). Nevertheless, the lap shear strength of the PUDAs are sufficient compared to the previously reported PSAs for flexible electronics [42].

The observed behavior is consistent with the adhesion–cohesion trade‐off commonly observed in pressure‐sensitive adhesives [42]. Increasing the PUDA content increased the gel fraction and effective crosslink density, enhancing the cohesive strength of the OCA network. However, excessive crosslinking restricted segmental mobility and substrate wetting, resulting in reduced adhesion performance. These findings highlight the importance of balancing network connectivity and molecular mobility in high‐performance OCA systems.

As high‐value, multifunctional display modules become thinner and more integrated, the need for clean and damage‐free reuse has become increasingly critical beyond sustainability considerations alone [46, 47]. From this perspective, clean removability after debonding is essential to enable recycling of the substrate without the need for additional cleaning steps. To evaluate this aspect, residue formation was examined after peel testing using scanning electron microscopy and energy‐dispersive X‐ray spectroscopy, as shown in Figure 2e. At room temperature, both the 0.175 PUDA and 0.175 HDDA samples showed no detectable residue on the surface after debonding. Importantly, the 0.175 PUDA maintained this residue‐free behavior even under high‐temperature conditions (80°C), where cohesive failure is more likely to occur [48]. In contrast, the 0.175 HDDA left behind clear adhesive residues remaining on the substrate, as confirmed by surface morphology and elemental mapping. These results demonstrate that the PUDA OCA enables clean and reliable debonding, making it particularly suitable for applications where substrate reuse and recyclability are required.

### 2.3 Optical Properties of OCAs

**FIGURE 3:**
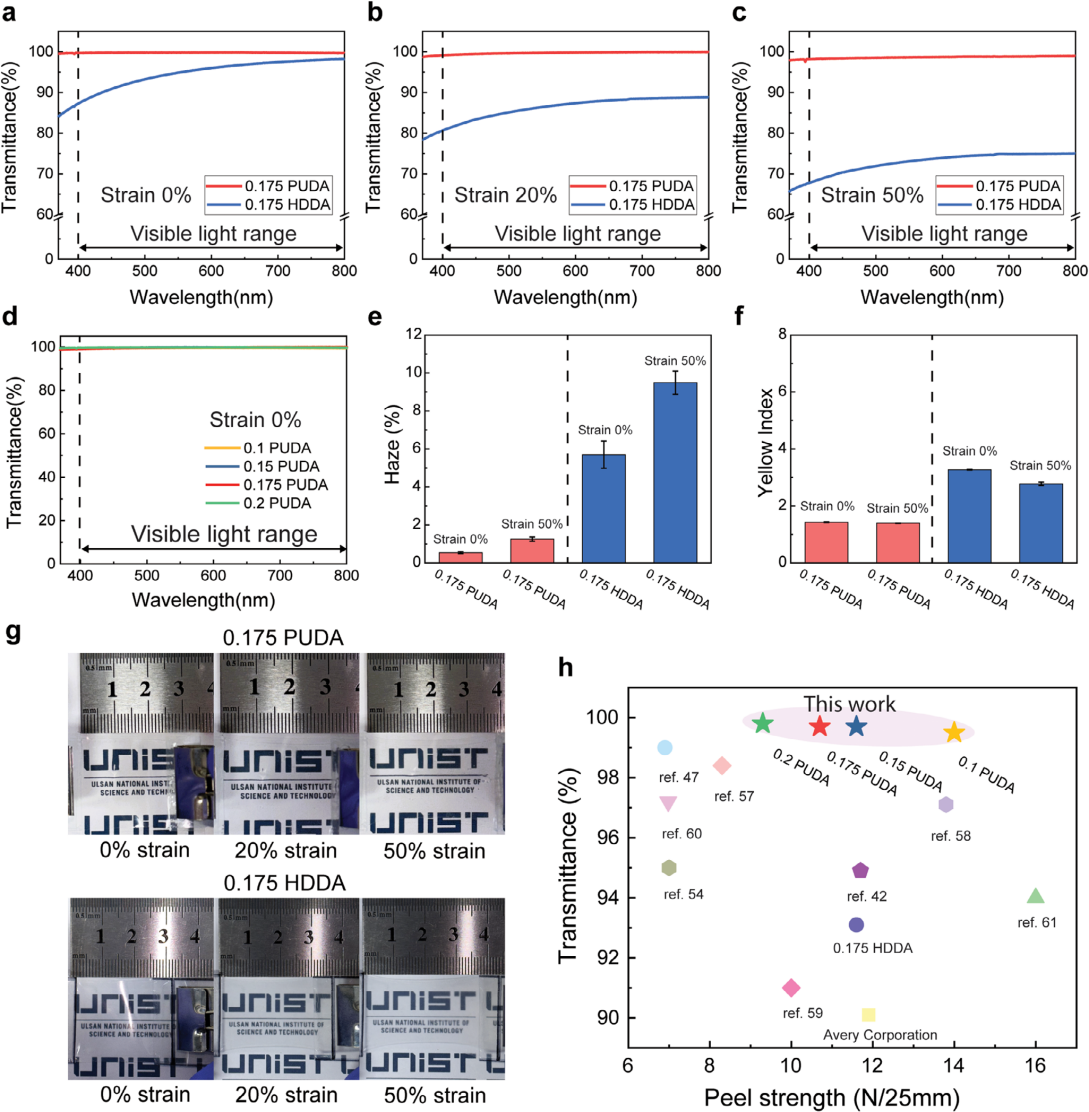
Optical transmittance of OCAs crosslinked with PUDA and HDDA. The transmittance of 0.175 PUDA and 0.175 HDDA under (a) 0% strain, (b) 20% strain, and (c) 50% strain. (d) The transmittance of PUDA OCAs with varying PUDA contents. (e) Haze values of OCAs (PUDA and HDDA) measured at 0% and 50% strain. (f) Yellow index values of OCAs (PUDA and HDDA) measured at 0% and 50% strain. (g) Optical images of 0.175 PUDA and 0.175 HDDA under different strain. (h) Comparison of optical transmittance and peel strength of PUDA crosslinked OCAs with previously reported OCAs and a commercial PSA (Avery Corporation).

OCAs are positioned directly along the optical path between the light‐emitting layer and the viewer, therefore maintaining high optical transmittance is extremely critical for display performance [4]. Figure 3 shows the optical properties of 0.175 PUDA and 0.175 HDDA. The optical transmittance of PUDA adhesive films was evaluated in the visible light range, and all samples exhibited exceptionally high transmittance exceeding 99.8% due to excellent intrinsic optical clarity of the PUDA, surpassing 0.175 HDDA and a commercial PSA (Figure S11).

From an energy‐efficiency perspective, the optical transmittance of the OCA directly influences OLED power consumption [6, 7, 8, 49]. The external luminance (*L_out_*) is determined by the internally generated luminance (*L_OLED_*) and the total transmittance of the display stack (*T_stack_*), including the OCA layer. To achieve a target luminance (*L_target_*), higher internal luminance is required as the stack transmittance decreases. Since OLED power consumption (*P*) scales with (*L_OLED_*), it is inversely proportional to the stack transmittance (*T_stack_*) [6, 7]:

$$P\propto \frac{1}{T_{stack}}$$

Under identical brightness conditions, the PUDA OCA (99.8% transmittance) consumes 7.31% lower OLED power than the HDDA OCA (93% transmittance) to achieve the same external luminance. Considering that the display typically accounts for approximately 50%, of the total power consumption in smartphones [50, 51], this improvement translates into an overall device‐level power reduction of approximately 3.66%. Importantly, this corresponds to a potential increase in total battery operating time of approximately 4%, highlighting that even small differences in OCA transmittance can produce meaningful improvements in practical device energy efficiency.

More importantly, PUDA crosslinked adhesives exhibited outstanding optical stability under mechanical deformation, maintaining high transparency even under strain. At 0% strain (Figure 3a), the 0.175 PUDA showed transmittance above 99.8% across the visible region (400–800 nm), whereas the 0.175 HDDA exhibited a lower value of 93%. Unlike previous reports where transmittance decreases due to semi‐crystallinity [52, 53], the PUDA showed less than a 1.2% reduction up to 50% strain, maintaining 99.6% at 20% strain (Figure 3b) and 98.6% at 50% strain (Figure 3c). In contrast, the 0.175 HDDA showed a significant decrease to 85.1% and 71.9% at 20% and 50% strain, respectively. These results demonstrate that the PUDA crosslinked network effectively preserves optical clarity under tensile deformation.

The optical transmittance of PUDA OCAs with varying PUDA contents remained consistently high across the visible region, exceeding 99.8%, indicating that incorporation of PUDA does not compromise intrinsic optical clarity (Figure 3d). In addition to optical transmittance, haze and yellow index were also systematically evaluated, as low haze is essential for minimizing light scattering and maintaining display sharpness [54, 55], while a low yellow index ensures minimal yellowing and reduced optical deterioration [56]. The haze values at 0% and 50% strain revealed a clear distinction between PUDA and HDDA (Figure 3e). For 0.175 PUDA, haze remained extremely low, increasing only slightly from 0.54% ± 0.06% at 0% strain to 1.25% ± 0.11% at 50% strain. In contrast, 0.175 HDDA exhibited significantly higher haze, increasing from 5.70% ± 0.71% to 9.49% ± 0.61%, indicating greater light scattering during deformation. The yellow index (YI) values under 0% and 50% strain are shown in Figure 3f. The 0.175 PUDA exhibited low YI values of 1.43 ± 0.02 at 0% strain and 1.39 ± 0.01 at 50% strain, while 0.175 HDDA showed higher values of 3.27 ± 0.01 and 2.78 ± 0.06 at 0% and 50% strain, respectively. The slight decrease in YI during tensile deformation should be attributed to thickness reduction of deformed OCAs.

Figure 3g shows optical images of 0.175 PUDA and 0.175 HDDA under 0%, 20%, and 50% strain. The 0.175 PUDA maintained visual transparency without noticeable whitening even at high strain, whereas the 0.175 HDDA exhibited visible whitening and optical degradation with increasing strain. Overall, these results demonstrate that PUDA maintains superior optical properties under mechanical strain compared to HDDA. Finally, Figure 3h compares the optical transmittance and peel strength of the PUDA OCAs with previously reported OCAs and a commercial PSA (Avery Corporation) [42, 47, 54, 57, 58, 59, 60, 61]. The PUDA OCAs are positioned in the upper region of the plot, achieving an ultra‐high transmittance (>99.8%) while maintaining competitive peel strength, demonstrating their outstanding potential for practical flexible display applications.

### 2.4 Strain Recovery Properties of OCAs

Rapid recovery of the original dimensions after mechanical deformation is a critical requirement for optically clear adhesives used in flexible display applications, since insufficient recovery under repeated strain can induce residual deformation and deterioration of optical and mechanical performance [3]. During repetitive deformation, the continuously applied strain can both disrupt and regenerate hydrogen bonds within the adhesive network [34]. The newly formed hydrogen bonds generated during straining hinder complete chain relaxation after stress release, resulting in prolonged recovery time and, in some cases, permanent deformation [34, 42].

**FIGURE 4:**
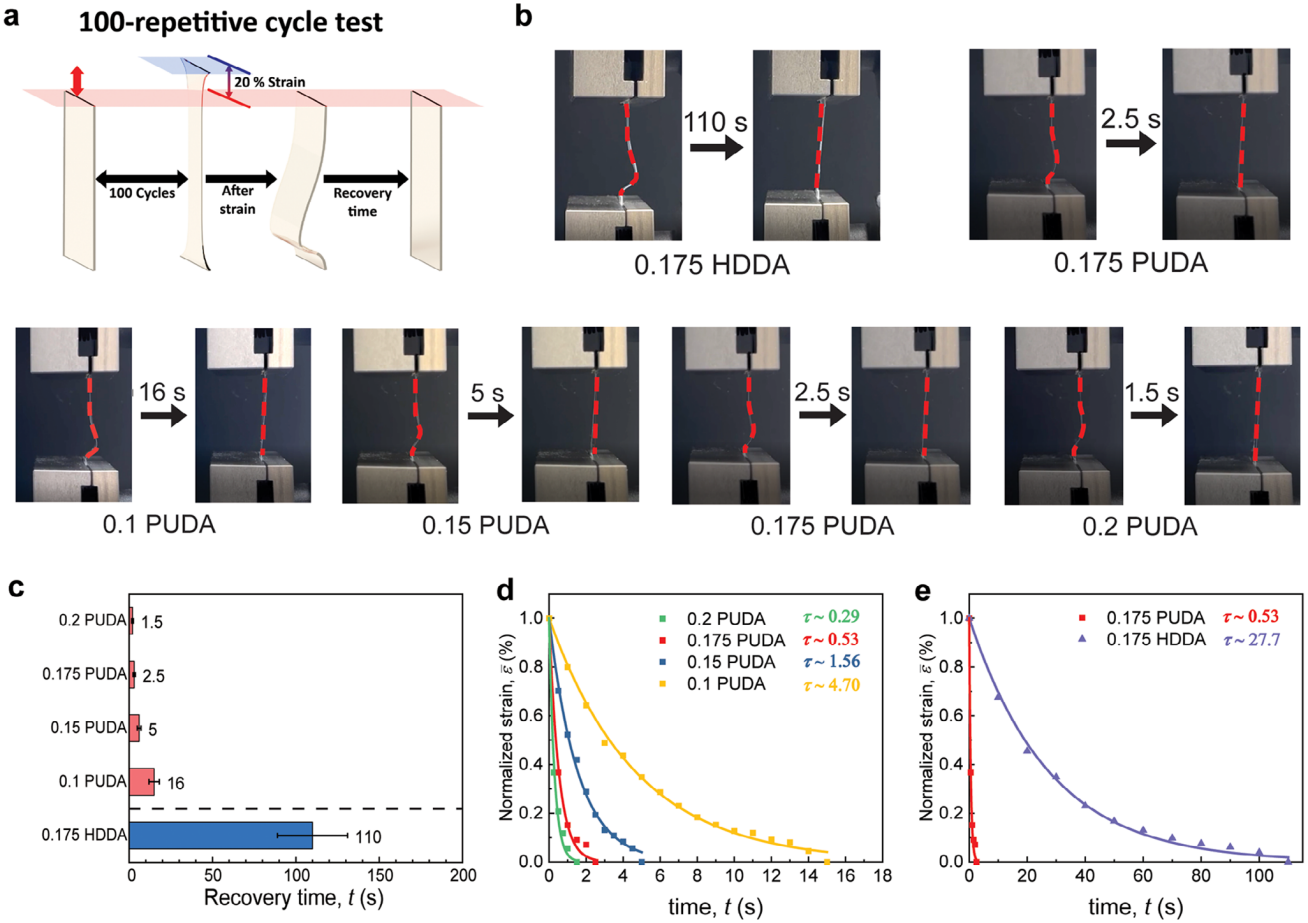
(a) Schematic illustration of the strain recovery after a 100‐cycle repetitive test. (b) Images showing the deformation of OCAs immediately after the 100‐cycle test, and after full recovery. (c) Full recovery time of OCAs and (d, e) strain recovery behavior analyzed by length monitoring and single‐exponential decay fitting to determine the recovery time constant (τ).

To evaluate the strain recovery behavior of the prepared OCAs, a 100‐cycle repetitive test was conducted under an applied strain of 20%, as shown in Figure 4a and Movies S1 and S2. Representative images obtained immediately after the 100th cycle and after full recovery are presented in Figure 4b, revealing a clear dependence of recovery behavior on PUDA content. As summarized in Figure 4c, the recovery time systematically decreased with increasing PUDA concentration. The 0.1 PUDA sample required 16 s to recover its original length, whereas 0.15 PUDA recovered within 5 s and 0.175 PUDA exhibited even faster recovery within 2.5 s. The most highly crosslinked formulation, 0.2 PUDA, showed the fastest recovery within 1.5 s. In contrast, the 0.175 HDDA exhibited markedly slower recovery, requiring more than 100 s for full recovery.

This pronounced difference originates from the distinct molecular characteristics of the PUDA crosslinked network. In particular, the cyclohexane ring in H_6_XDI incorporated into PUDA interrupts the formation of hydrogen bonds between urethane groups and polymer chains, as previously reported by FT‐IR analyses [62]. In addition, the PEG‐containing segments in PUDA act as soft domains with excellent flexibility [63], resulting in high mobility of the polymer chains. Moreover, the relatively high molecular weight of PUDA increases the interchain spacing, thereby hindering the formation of strain‐induced hydrogen bonds between neighboring CEA units during cyclic deformation [42, 64].

Furthermore, increasing PUDA content enhances the crosslinking density of the adhesive network, leading to a reinforced elastic structure with improved restoring force. Although higher crosslinking density partially restricts chain mobility, it effectively suppresses the generation of newly formed hydrogen bonds during deformation. As a result, rapid and stable strain recovery is achieved. This interpretation is further supported by the hysteresis loss behavior observed during cyclic loading (Figure S12). As summarized in Table S6, the hysteresis loss decreased from 5.16 kJ m^−^^3^ for 0.1 PUDA to 3.80 kJ m^−^^3^ for 0.2 PUDA. In contrast, the 0.175 HDDA sample exhibited a higher hysteresis loss (4.76 kJ m^−^^3^), which is consistent with its slower recovery behavior.

To quantitatively evaluate the recovery kinetics, the time‐dependent strain recovery behavior was monitored following the removal of applied stress. The recovery curves (Figure 4d,e) were fitted using a normalized single‐exponential decay model, expressed as

$$\bar{\varepsilon }=\exp\left(-t/\tau \right)$$

where τ represents the recovery time constant. All OCAs were well fitted with high coefficients of determination (R^2^ ≈ 0.99; Table S7), indicating that the recovery process can be described by a dominant relaxation mechanism. The τ values decreased significantly with increasing PUDA content, from 4.70 ± 0.07 s for 0.1 PUDA to 0.28 ± 0.02 s for 0.2 PUDA, reflecting rapid chain reorganization and faster strain recovery. In contrast, HDDA crosslinked OCA exhibited a much larger τ value (27.68 ± 0.60 s), confirming substantially slower recovery. These quantitative results are in good agreement with the directly measured recovery times and further support the rapid elastic recovery of PUDA OCAs.

Irreversible deformation remaining after stress removal under sustained loading can accumulate during repeated folding and bending, leading to wrinkles or optical defects in display modules [3]. Therefore, a high creep recovery ratio is a critical parameter for optically clear adhesives, as it minimizes permanent deformation and ensures long‐term stability [3, 65]. To evaluate the recovery performance of the OCAs under constant stress conditions, creep and recovery tests were conducted at an applied stress of 1000 Pa, and the normalized creep–recovery curves are shown in Figure S13. PUDA OCAs exhibited rapid strain recovery immediately after load removal. Even after a relatively long creep duration of 300 s, high creep recovery ratios of 86.1%, 87.8%, 88.3%, and 88.8% were maintained for 0.1, 0.15, 0.175, and 0.2 PUDA, respectively. (Table S8) These high creep recovery ratios indicate minimal residual deformation and strong resistance to irreversible deformation under long‐term loading [66]. In contrast, 0.175 HDDA showed a significantly lower recovery ratio of 74.6%.

Overall, the combination of short recovery times (down to 1.5 s), low hysteresis loss (≈3.8 kJ m^−^^3^), small recovery time constants with high fitting reliability (τ ≈ 0.28 s, R^2^ ≈ 0.99), and high creep recovery ratios (up to 88.8% after 300 s) demonstrates that PUDA‐based OCAs exhibit outstanding recovery performance. These characteristics make PUDA crosslinked OCAs particularly suitable for flexible and foldable display applications that require repeated mechanical deformation without permanent damage or loss of performance.

**FIGURE 5:**
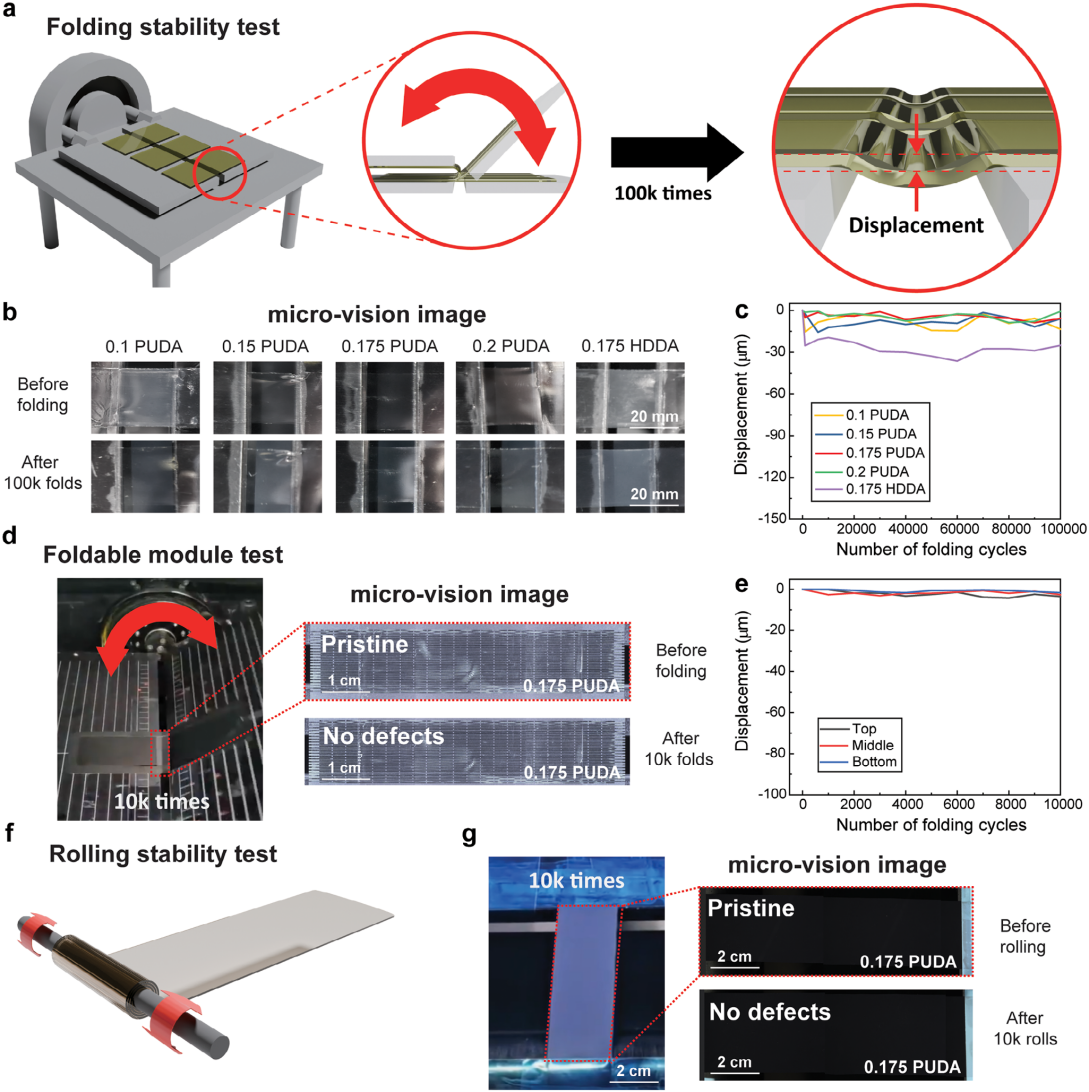
Folding and rolling stability test of OCAs. (a) Schematic of folding stability test and the displacement change of the OCA after folding. (b) Micro‐vision images of 0.1, 0.15, 0.175, and 0.2 PUDA, and 0.175 HDDA before and after 100 k folds. (c) A graph illustrating the displacement change of each OCA during 100 k folds. (d) Folding durability test on a foldable module with 0.175 PUDA, and micro‐vision images before and after 10 k folds. (e) Displacement at the top, middle, and bottom positions during 10 k folds. (f) Schematic of the rolling stability test. (g) Micro‐vision images of 0.175 PUDA before and after 10 k rolls.

The folding stability of the OCAs was systematically evaluated under repeated bending conditions with a curvature radius of 2 mm and up to 100 000 (100k) folding cycles to investigate their mechanical durability relevant to foldable display applications (Figure 5a). The sagging‐induced displacement was continuously monitored as a function of folding cycles while simultaneously capturing micro‐vision images. As shown in Figure 5b and Figure S14, PUDA OCAs exhibited stable displacement behavior without progressive accumulation of permanent deformation. After 100 k folding cycles, the displacement values of 0.1, 0.15, 0.175, and 0.2 PUDA were −13.41, −5.97, −5.68, and −0.72 µm, respectively, whereas the 0.175 HDDA sample showed a significantly larger displacement of −25.07 µm (Figure 5c and Table S9).

During folding, the OCA layer experiences simultaneous tensile and compressive stresses on the outer and inner surfaces, respectively, resulting in shear deformation within the adhesive layer [67]. The bending stress applied during folding is expressed as

$$\sigma =\left(E/\rho \right)\gamma$$

where 𝐸 is the elastic modulus, ρ is the radius of curvature, and γ is the distance from the neutral plane [67]. Furthermore, the bending strain applied to the outer and inner surfaces of the OCA can be expressed as

ε=γ/ρ

Under the present folding conditions, each OCA layer undergoes tensile and compressive strains of approximately ±2.5% on the outer and inner surfaces, respectively [67]. As the PUDA content increased from 0.1 to 0.175 mol%, the elastic modulus increased accordingly, leading to a gradual increase in the maximum bending stress during folding. As summarized in Table S10, the maximum bending stress *σ_max_* values increased from 2.00 kPa (0.1 PUDA) to 2.40 kPa (0.15 PUDA) and further to 3.23 kPa (0.175 PUDA). However, at 0.2 PUDA, *σ_max_* slightly decreased to 2.60 kPa, reflecting the lower *E* due to incomplete crosslinking [32, 68]. Notably, the differences in σ_max_ among the PUDA samples remain relatively small, and even at 0.175 PUDA, the bending stress is still remarkably low. In contrast, the 0.175 HDDA exhibited a significantly higher *σ_max_* value of 11.40 kPa, which is approximately four to five times greater than those of the PUDA OCAs.

Such low bending stress effectively reduces the mechanical load imposed on the adhesive layer during folding, thereby alleviating stress concentration and suppressing mechanical damage [69]. In particular, under repetitive folding conditions, localized stress accumulation is a primary driving force for crack initiation, interfacial delamination, and residual deformation [4]. The low bending stress of PUDA OCAs helps mitigate these failure pathways and contributes to improved long‐term reliability [70].

Furthermore, foldable module tests were conducted using an actual flexible display module (Figure 5d and Movie S3). The optimized 0.175 PUDA was laminated onto the module and subjected to 10 k folding cycles. The displacement values measured after 10 k cycles were −3.5 µm (top), −2.6 µm (middle), and −1.6 µm (bottom), demonstrating good dimensional stability across the folded region (Figure 5e and Table S11). Also, as shown in Figure S15, the micro‐vision images acquired before and after the folding tests showed no noticeable deformation or visible defects, such as cracks, wrinkles, or delamination, confirming that the surface condition was well maintained during repeated folding.

In addition, rolling stability tests were performed under continuous rolling deformation with a curvature radius of 20 mm (20R) at a rolling speed of 5 s per cycle for up to 10 000 cycles to evaluate the mechanical durability of the OCA (Figure 5f and Movie S4). The rolling test specimen was prepared by laminating the OCA between two polyurethane (PU) films (Figure S16). After 10 k rolling cycles, 0.175 PUDA maintained their original surface condition without observable defects, further confirming their mechanical robustness and recovery capability under practical operating conditions (Figure 5g and Figure S17). Collectively, the low bending stress, suppressed permanent deformation, and stable surface condition demonstrate that PUDA OCAs are mechanically more suitable than HDDA OCA for foldable and rollable display applications.

### 2.5 Rheological Properties and Stress Relaxation Properties of OCAs

Flexible and foldable displays consist of multiple functional layers that must deform together during bending [4]. Because each layer possesses a different stiffness, mechanical mismatch can generate localized stress concentrations, which may lead to cracking or delamination within the device stack [71]. Optically clear adhesives play a critical role in mitigating these issues by providing a soft interlayer that accommodates deformation and buffers the stress transferred to rigid components [67]. Accordingly, maintaining a sufficiently low modulus in OCA is essential to ensure mechanical stability under repeated folding.

The viscoelastic properties of the PUDA OCAs were investigated using a rheometer under frequency sweep conditions (Figure S8). The storage modulus of the OCAs increased with increasing PUDA crosslinker content, reflecting the enhanced network cohesion induced by higher crosslinking density. Nevertheless, all PUDA samples maintained a sufficiently low storage modulus across the measured frequency range. For flexible and foldable display applications, the storage modulus of OCAs should be lower than 0.3 MPa to effectively relax applied stress by tuning the neutral plane during bending [29, 67]. As shown in Figure S18, the PUDA OCAs are well positioned within the PSA region, satisfying the Dahlquist criterion (G′ < 0.1 MPa at 1 Hz) [72]. Notably, the storage modulus of all PUDA crosslinked OCAs was even lower than 0.1 MPa, indicating their strong capability to accommodate deformation and effectively relieve mechanical stress under folding conditions.

Low‐modulus OCAs facilitate polymer chain flow and rearrangement, enabling efficient dissipation of applied stress during mechanical deformation [66, 71]. Therefore, high stress relaxation capability is a critical requirement for flexible display adhesives to prevent stress accumulation under repeated folding [13]. The stress relaxation behavior was evaluated by monitoring the time‐dependent decay of normalized stress under a constant strain of 20% (Figure S19). As the PUDA content increased, the stress relaxation ratios gradually decreased from 58.5% to 54.2%, 41.9%, and 37.3%, respectively. (Table S12) This trend indicates that increasing crosslink density suppresses stress relaxation by restricting chain rearrangement and segmental motion, while still maintaining a mechanically stable network suitable for flexible display applications.

## 3 Conclusion

In summary, we synthesized a polyurethane diacrylate (PUDA) crosslinker derived from biomass‐based isosorbide and H_6_XDI to develop optically clear adhesives (OCAs) with high optical transparency and superior mechanical recovery. Among the investigated compositions, the OCA containing 0.175 mol% PUDA exhibited the most optimized performance. It achieved exceptional optical transmittance of 99.8% in the visible region and maintained 98.6% even under 50% tensile strain. The optimized formulation also showed stable adhesion comparable to commercial adhesives with clean, residue‐free debonding after 80°C heating. Mechanically, 0.175 PUDA exhibited an extremely low modulus (G'∼0.029 MPa at 1 Hz), leading to a significantly reduced maximum bending stress (σ_max_) during deformation. It also demonstrated a high creep recovery ratio (88.3%), indicating minimal residual deformation after mechanical loading. In addition, PUDA showed rapid recovery behavior of approximately 2.5 s, and no defects were observed during folding and rolling stability tests. Overall, PUDA incorporated OCAs maintain high transparency and fast recovery under folding and tensile stress conditions, demonstrating potential for flexible and stretchable electronic applications.

## 4 Experimental Section

### 4.1 Materials

Isosorbide‐polyol was supplied by PUCORE corporation (Korea) and 1,3‐bis(isocyanatomethyl)cyclohexane (H_6_XDI) was purchased from TCI Chemicals. 2‐Carboxyethyl acrylate (CEA) and 2‐ethylhexyl acrylate (EHA) were obtained from Sigma‐Aldrich and used as acrylate monomers. Hydrophobic fumed silica (Aerosil R974) was purchased from Evonik Industries. 1,6‐Hexanediol diacrylate (HDDA) was purchased from Alfa Aesar and used as a reference crosslinker. Phenylbis(2,4,6‐trimethyl‐benzoyl) phosphine oxide (photoinitiator) and dibutyltin dilaurate (DBTDL, catalyst) were purchased from TCI Chemicals. Poly (ethylene terephthalate) (PET) film (SH82) and releasing film (SG31) were purchased from SKC. All reagents were used without further purification. As a reference, the commercial PSA (Avery Dennison, USA) was used.

### 4.2 Synthesis of Polyurethane Diacrylate (PUDA)

PUDA crosslinkers were synthesized via a two‐step urethane and acrylation reaction. In a dried two‐neck round‐bottom flask equipped with a mechanical stirrer and nitrogen inlet, H_6_XDI (5.8 g, 30 mmol) and isosorbide‐polyol (50.0 g, 25 mmol) were charged in EHA (45 ml) under a nitrogen atmosphere and reacted at 60°C for 2 h. DBTDL (79 mg) was added as a catalyst, and the mixture was stirred until the NCO peak of H_6_XDI (≈2264 cm^−^^1^) in the FT‐IR spectrum disappeared, confirming the formation of urethane linkages. After completion of the urethane reaction, 2‐hydroxyethyl methacrylate (1.2 g, 9 mmol, 0.3 equiv. relative to the initial H_6_XDI feed) was added as an end‐capping agent to convert the remaining terminal NCO groups into methacrylate functionalities. The reaction was continued for an additional 0.5 h until no residual isocyanate signal was observed in the FT‐IR spectrum (Figure S2), confirming complete end‐capping of the terminal NCO groups. The resulting PUDA was obtained as a solution with a solid content of 60 wt.% in EHA and was used directly for OCA formulation without further purification or isolation. The molecular weight (Mw) of the synthesized PUDA was determined by gel permeation chromatography to be approximately 44 000 g mol^−^^1^ (Figure S3 and Table S1).

### 4.3 Characterization of Synthesized PUDA

The chemical structures of PUDA were analyzed by FT‐IR and ^1^H NMR. ^1^H NMR spectrum of PUDA was recorded using a 400 MHz NMR spectrometer (AVANCE III HD, Bruker, USA) with CDCl_3_ as the solvent. FT‐IR spectra were performed on FT/IR‐4600 (JASCO, Japan) with attenuated total reflection mode. The weight‐average molecular weight (Mw) and molecular weight distribution of PUDA were determined by GPC (1200S, Agilent, USA) using tetrahydrofuran (THF) as the eluent and polystyrene standards for calibration.

### 4.4 Preparation of OCA Films

OCA formulations were prepared by mixing CEA and EHA in a 4:6 molar ratio. PUDA or HDDA was then added as a crosslinker at various contents (0.1, 0.15, 0.175, and 0.2 mol% relative to total monomer), and phenylbis(2,4,6‐trimethyl‐benzoyl) phosphine oxide (0.1 mol%) was introduced as a photoinitiator. The mixtures were stirred thoroughly using a vortex mixer until homogeneous. The resulting syrups were coated onto PET substrates using a knife coater to obtain wet films corresponding to a final thickness of 100 ± 10 µm after curing. A release film was laminated on top of the coated layer, and the samples were cured under UV irradiation (365 nm) using a conveyor‐type curing system. After curing, the OCA films were stored in the dark at room temperature prior to testing.

### 4.5 Gel Fraction of OCAs

To determine the gel fraction, cured OCA films were cut into small pieces (≈10 × 10 mm), dried at 40°C under vacuum, and weighed to obtain the initial mass (*W*_0_). The samples were then immersed in excess toluene at room temperature for 24 h to extract the soluble fraction. After extraction, the samples were removed, gently blotted to remove surface solvent, and dried again in a vacuum oven at 40°C for 24 h until a constant mass (*W_g_*) was reached. The gel fraction was calculated as:

$\mathrm{Gel}\;\mathrm{fraction}\;(\%)\;=\frac{W_{g}}{W_{0}}\;\times 100$

### 4.6 Mechanical Properties

Tensile properties of the OCA films were measured using a UTM (WL2100C, Withlab, South Korea) at room temperature. Rectangular specimens were prepared with dimensions suitable for uniaxial tensile testing, and the crosshead speed was set to 100 mm min^−1^. Stress–strain curves were recorded, and Young's modulus, ultimate tensile strength (UTS), elongation at break, and toughness were obtained from the curves. Each condition was tested at least five times to ensure reproducibility. Rheological properties were measured with a rheometer (Kinexus pro+, NETZSCH, Germany).

### 4.7 Thermal Properties

DSC (Q200, TA Instrument, USA) was used to determine the glass transition temperature (*T*_g_) of the OCA films. Samples were placed in platinum pans and analyzed from −80°C to 80°C at a heating rate of 10°C min^−^^1^ under nitrogen. All *T*_g_ values discussed in this manuscript were obtained from the 2nd heating scan and determined from the midpoint of the corresponding heat capacity change. Thermal stability of the cured OCAs was evaluated by TGA (Q400, TA Instrument, USA). The specimens were heated from 40°C to 600°C at a rate of 10°C min^−^^1^ under nitrogen, and the onset and main decomposition temperatures were recorded.

### 4.8 Adhesion Properties

Peel adhesion tests were carried out to evaluate the adhesion performance of the OCA films on various substrates. OCA strips (25 × 200 mm) were laminated onto stainless steel (SUS304), glass, PMMA, and PET substrates using a 2.5 kg hand roller, passed twice at a speed of 10 mm s^−^^1^. The laminated samples were conditioned at room temperature for 15 min before testing. 180° peel tests were performed at a crosshead speed of 300 mm min^−^^1^ using a UTM (WL2100C, Withlab, South Korea). Peel strength measurements were performed at room temperature in a 180° configuration with a crosshead speed of 300 mm min^−^^1^ (ASTM D3330). To assess the temperature‐dependent adhesion behavior, additional 180° peel tests were conducted at −20°C, 0°C, and 60°C using a chamber‐type UTM (QM200EL, QMESYS, South Korea). The lap shear test was performed at room temperature with a crosshead speed of 1.3 mm min^−^^1^ in accordance with ASTM D1002. Probe tack measurements were carried out using a texture analyzer (TXA, Yeonjin, South Korea) equipped with a flat‐type stainless‐steel cylindrical probe (5 mm in diameter). Following ASTM D2979, the probe was brought into contact with the OCA under a 100 gf load for 1 s, and both the approach and withdrawal were performed at a constant speed of 10 mm s^−^^1^. For all adhesion measurements, including 180° peel tests at room temperature and at varying temperatures (−20°C, 0°C, and 60°C), peel tests on different substrates, lap shear tests, and probe tack tests, each sample was tested five times (*n* = 5) to confirm repeatability, and the results are reported as mean ± standard deviation with error bars representing the standard deviation. SEM imaging and EDS mapping were conducted using an SU7000 electron microscope (Hitachi High‐Technologies) operated at 10 kV to examine adhesive residue on SUS substrates. Residue analysis of the 0.175 PUDA and 0.175 HDDA sample was performed after adhesion at 80°C for 5 min, followed by an additional 10 min at room temperature before detachment.

### 4.9 Recovery Properties

The recovery behavior of the OCAs was systematically investigated through cyclic mechanical testing. Repetitive tensile loading–unloading experiments were carried out using a universal testing machine (UTM, DAO‐u1, DAO Technology, South Korea) at room temperature. Rectangular OCA specimens with dimensions of 13 × 39 mm^2^ (W × L) were subjected to 100 consecutive stretching–recovery cycles at a crosshead speed of 500 mm min^−^^1^ under a fixed strain of 20%. All final measurements were based on five repetitions (*n* = 5), and the reported values are presented as mean ± standard deviation to ensure reliability. The energy dissipation during cyclic deformation was quantified by calculating the hysteresis loss from the enclosed area of the stress–strain curve obtained in the 100th cycle.

Following the completion of cyclic testing, the strain recovery kinetics of the OCAs were evaluated. After releasing the applied load, the length of each sample at 0% nominal strain was continuously monitored until the material returned to its original length, defined as the point at which no further dimensional change was observed. To enable quantitative comparison, the measured strain values were normalized using Min‐Max normalization ($\bar{\varepsilon }$= (*ε* – *ε*_min_) (*ε*_max_ – *ε*_min_)^−1^). The normalized recovery curves were subsequently fitted with a single‐exponential decay function, and the corresponding time constants (τ) were extracted.

The folding recovery performance of the OCAs was further evaluated under repeated bending conditions. Folding tests were conducted using a folding machine (Foldy‐200, FlexiGo, South Korea), where the OCA films were attached to a bending radius of 2 mm (2R). An in‐folding mode was employed at a folding speed of 1 cycle s^−^^1^ for up to 100 000 folding cycles at room temperature. Optical micro‐vision images were collected before and after the folding test, and displacement during folding was recorded at 1000, 5000, and 10 000 cycles, followed by measurements at every subsequent interval of 10 000 cycles.

The rolling test specimen was prepared by laminating the OCA between two polyurethane (PU) films, where the adhesive layer was first attached onto a PU film and subsequently covered with another PU film (Figure S15). The rolling durability performance of the OCA (0.175 PUDA) was further evaluated under repeated rolling conditions. Rolling tests were conducted using a rolling test machine (Rolly&Slidy‐20, FlexiGo, South Korea) with a bending radius of 20 mm (20R) at a rolling speed of 5 s per cycle at room temperature. One cycle sequence consisted of 1000 rolling cycles at room temperature, followed by an inspection at the same temperature. This sequence was repeated ten times, resulting in a total of 10 000 rolling cycles. Optical micro‐vision images were collected before and after the rolling test to examine whether any defects were generated in the OCA film.

### 4.10 Stress Relaxation Properties

The stress relaxation characteristics of the OCAs were examined using a rotational rheometer (Kinexus Pro+, NETZSCH, Germany). Disk‐shaped samples with a diameter of 20 mm and a thickness of 100 µm were prepared following the same procedure used for rheological measurements. Stress relaxation tests were performed by imposing a constant strain of 20% and monitoring the temporal decay of stress over a duration of 10 min at room temperature.

### 4.11 Optical Properties

The optical transmittance of the OCA films was measured using a UV‐Vis‐NIR spectroscopy (V‐750, JASCO, Japan). OCA films with a thickness of 100 µm were laminated onto bare glass (Slide Glass, Marienfeld, Germany) and evaluated at strains of 0%, 20%, and 50% using a custom stretching setup. The transmittance at 800 nm was used as a reference, and the spectra were recorded in the wavelength range of 370–800 nm to assess changes in optical clarity under deformation. In addition, the haze and yellowness index (YI) of the OCA films were measured using a spectrophotometer (CM‐3700A, Konica Minolta, Japan). For haze and YI measurements, OCA films were laminated onto MATSUNAMI MICRO SLIDE GLASS (S9213, Japan). Each sample was independently measured five times (*n* = 5), and all reported values are presented as mean ± standard deviation.

## Author Contributions

**Dong Woo Kim**: formal analysis. **Dahyun Jin**: formal analysis. **Jinhoon Lee**: formal analysis. **Dong Woog Lee**: conceptualization, funding acquisition, writing – review and editing, supervision, methodology, project administration. **Ki Sung Jung**: formal analysis, resources. **Yeonseo Kim**: formal analysis. **Geonwoo Lee**: formal analysis. **Myung‐Jin Baek**: conceptualization, methodology, investigation, project administration, visualization, writing – review and editing. **Yoonji Choi**: methodology, investigation, data curation, formal analysis, writing – original draft, visualization.

## Conflicts of Interest

The authors declare no conflicts of interest.

## Supporting information




**Supporting File 1**: advs77348‐sup‐0001‐SuppMat.docx.

**Supporting File 2**: advs77348‐sup‐0002‐MovieS1.mp4.

**Supporting File 3**: advs77348‐sup‐0003‐MovieS2.mp4.

**Supporting File 4**: advs77348‐sup‐0004‐MovieS3.mp4.

**Supporting File 5**: advs77348‐sup‐0005‐MovieS4.mp4.

## Data Availability

The data that support the findings of this study are available in the supplementary material of this article.
