# Supplementary material for: Isosorbide‐Based Optically Clear Adhesives With Ultrahigh Transparency and Rapid Strain Recovery for Flexible Displays
Source: Adv Sci (Weinh). 2026 Aug 30:e77348. Online ahead of print. doi: 10.1002/advs.77348 (PMC13526423; doi:10.1002/advs.77348)
Supplement: Supplementary file 1 [file ADVS-9999-e77348-s003.docx]

**Supporting Information**

**Isosorbide-Based Optically Clear Adhesives with Ultrahigh Transparency and Rapid Strain Recovery for Flexible Displays**

*Yoonji Choi, Geonwoo Lee, Yeonseo Kim, Dahyun Jin, Jinhoon Lee, Ki Sung Jung, Dong Woo Kim, Myung-Jin Baek*, Dong Woog Lee**

Y. Choi, G. Lee, Y. Kim, D. Jin, J. Lee, M.-J. Baek, D. W. Lee

School of Energy and Chemical Engineering, Ulsan National Institute of Science and Technology (UNIST), 50 UNIST-gil, Ulsan, 44919, Republic of Korea

* E-mail: bagmj100@unist.ac.kr; dongwoog.lee@unist.ac.kr

K. S. Jung, D. W. Kim

42, Gunpocheomdansaneop 2-ro, Gunpo-si, Gyeonggi-do, Republic of Korea

* E-mail: keh@youngwoo.com; qwer113@youngwoo.com

**Figure S1.** ^1^H NMR spectra of Polyurethane diacrylate (PUDA).

**Figure S2.** FT-IR spectra of PUDA.

**Figure S3.** The molecular weight distribution curves of synthesized PUDA crosslinker.

**Figure S4.** The peel adhesion strength of 0.175 PUDA with different monomer ratios.

**Figure S5.** DSC thermograms (2^nd^ heating scan) showing glass transition temperature (*T*_g_s) of OCAs with different crosslinkers (PUDA, HDDA) and different PUDA ratios.

**Figure S6.** TGA thermograms showing 50% degradation temperature (*T*_50d_) of OCAs crosslinked with different crosslinkers (PUDA, HDDA) and different PUDA ratios.

**Figure S7.** Mechanical properties of OCAs. Stress-strain curves of OCAs (a) with varying PUDA contents and (b) crosslinked with different crosslinkers (PUDA, HDDA).

**Figure S8.** Rheological properties of the OCAs. Storage modulus of OCAs (a) with varying PUDA contents and (b) crosslinked with different crosslinkers (PUDA, HDDA). Loss modulus of OCAs (c) with varying PUDA contents and (d) crosslinked with different crosslinkers (PUDA, HDDA).

 **Figure S9.** Temperature-dependent peel strength of (a) 0.175 PUDA and (b) 0.175 HDDA OCA measured at -20, 0, 25, and 60 °C.

**Figure S10.** Rheological properties of the OCAs. Storage modulus of OCAs at (a) -5 °C and (b) 40 °C.

**Figure S11**. Transmittance of OCAs crosslinked with different crosslinkers (PUDA, HDDA) and commercial PSA (Avery Corporation).

**Figure S12.** 100-repetitive cycle test of OCAs. Stress–strain curve of the 100-repetitive cycle test of (a) 0.1 PUDA, (b) 0.15 PUDA, (c) 0.175 PUDA, (d) 0.2 PUDA, and (e) 0.175 HDDA. (f) Stress–strain curve at the 100^th^ cycle of 0.1 PUDA, 0.15 PUDA, 0.175 PUDA, 0.2 PUDA and 0.175 HDDA.

**Figure S13.** Normalized creep and recovery curve (σ = 1000 Pa) of OCAs (a) with varying PUDA contents, (b) crosslinked with different crosslinkers (PUDA, HDDA) and (c) comparison with commercial PSA (Avery Corporation).

**Figure S14.** Optical microscopy images of OCAs during 100,000 folds.

**Figure S15.** Optical microscopy images of 0.175 PUDA during 10,000 folds on a foldable module.

**Figure S16.** Schematic illustration of the rolling test specimen consisting of an OCA layer laminated between two PU films.

**Figure S17.** Optical microscopy images of 0.175 PUDA during 10,000 rolls.

**Figure S18.** Chang’s window showing the viscoelastic property distribution (G′ vs G″) of the prepared OCAs relative to the Dahlquist criterion.

**Figure S19.** Normalized stress – time curve of the stress relaxation test of OCAs (a) with varying PUDA contents and (b) crosslinked with different crosslinkers (PUDA, HDDA).

**Table S1.** The molecular weights and polydispersity index (PDI) of synthesized crosslinker.

|  | **PUDA** |
| --- | --- |
| ***M*_n_ [g mol^-1^]** | 27,000 |
| ***M*_w_ [g mol^-1^]** | 44,000 |
| **PDI** | 1.62 |

**Table S2.** The gel fraction of synthesized OCAs with different crosslinker (PUDA, HDDA) and different PUDA ratios.

|  | **0.1 PUDA** | **0.15 PUDA** | **0.175 PUDA** | | **0.2 PUDA** | **0.175 HDDA** | |
| --- | --- | --- | --- | --- | --- | --- | --- |
| **Gel fraction [%]** | 67 | 80.3 | | 84.9 | 86.3 | | 81.4 |

**Table S3.** Mechanical properties of OCAs with different crosslinkers (PUDA, HDDA) and different PUDA ratios.

|  | **0.1 PUDA** | | | **0.15 PUDA** | | **0.175 PUDA** | **0.2 PUDA** | **0.175 HDDA** |
| --- | --- | --- | --- | --- | --- | --- | --- | --- |
| **Young’s modulus**  **[kPa]** | | 96.2 ± 25.5 | 110.1 ± 6.9 | | | 134.0 ± 8.2 | 118.6 ± 20.2 | 497.5 ± 37.5 |
| **Toughness**  **[MJ m^-3^]** | | 1.9 ± 0.1 | | | 2.3 ± 0.2 | 2.4 ± 0.2 | 1.5 ± 0.1 | 4.9 ± 1.2 |
| **Ultimate tensile strength**  **[MPa]** | | 0.5 ± 0.1 | | | 0.7 ± 0.2 | 0.8 ± 0.1 | 0.4 ± 0.1 | 2.1 ± 0.3 |
| **Elongation at break**  **[%]** | | 933.8 ± 59.0 | | | 918.2 ± 44.3 | 882.3 ± 43.9 | 775.3 ± 34.7 | 583.0 ± 61.8 |

**Table S4.** The peel strength, lap shear and probe tack of OCAs with different crosslinkers (PUDA, HDDA) and different PUDA ratios.

|  | **Peel strength**  **[N 25 mm^-1^]** | **Lap shear**  **[kPa]** | **Probe tack**  **[kPa]** |
| --- | --- | --- | --- |
| **0.1 PUDA** | 13.9 ± 1.4 | 85.7 ± 2.9 | 202 ± 21 |
| **0.15 PUDA** | 11.6 ± 0.9 | 60.8 ± 4.5 | 200 ± 15 |
| **0.175 PUDA** | 10.7 ± 0.5 | 59.8 ± 6.6 | 197 ± 9 |
| **0.2 PUDA** | 9.3 ± 0.4 | 56.8 ± 5.2 | 184 ± 7 |
| **0.175 HDDA** | 11.6 ± 1.0 | 147.4 ± 1.4 | 200 ± 26 |

**Table S5.** The peel strength of 0.175 PUDA on different substrates.

| **Substrates** | **Peel strength [N 25 mm^-1^]** |
| --- | --- |
| **SUS** | 10.7 ± 0.8 |
| **Glass** | 14.5 ± 1.7 |
| **PMMA** | 17.1 ± 0.8 |
| **PET** | 10.2 ± 0.8 |
| **PP** | 5.1 ± 0.3 |
| **Teflon** | 0.09 ± 0.03 |

**Table S6.** The hysteresis loss of 0.1 PUDA, 0.15 PUDA, 0.175 PUDA, 0.2 PUDA and 0.175 HDDA calculated by integrating the 100^th^ cycle curve area.

|  | **Hysteresis loss [kJ m^-3^]** |
| --- | --- |
| **0.1 PUDA** | 5.16 |
| **0.15 PUDA** | 4.05 |
| **0.175 PUDA** | 3.81 |
| **0.2 PUDA** | 3.80 |
| **0.175 HDDA** | 4.76 |

**Table S7.** Fitting parameter of the strain recovery of OCAs with PUDA and HDDA with normalized single exponential fitting.

|  | **0.1 PUDA** | **0.15 PUDA** | **0.175 PUDA** | **0.2 PUDA** | **0.175 HDDA** |
| --- | --- | --- | --- | --- | --- |
| ***ε_min_* [%]** | 0 | 0 | 0 | 0 | 0 |
| ***ε_max_* [%]** | 4.80 | 4.68 | 3.73 | 3.52 | 6.80 |
| ***τ*** | 4.70 ± 0.07 | 1.56 ± 0.03 | 0.53 ± 0.03 | 0.28 ± 0.02 | 27.68 ± 0.60 |
| **R-square** | 0.996 | 0.996 | 0.995 | 0.991 | 0.996 |

**Table S8.** Creep recovery ratio of OCAs with different crosslinkers (PUDA, HDDA) and different PUDA ratios.

|  | **0.1 PUDA** | **0.15 PUDA** | **0.175 PUDA** | **0.2 PUDA** | **0.175 HDDA** | **Avery** |
| --- | --- | --- | --- | --- | --- | --- |
| **Creep recovery ratio [%]** | 86.1 | 87.8 | 88.3 | 88.8 | 74.6 | 70.2 |

**Table S9.** The displacement changes of OCAs with different crosslinkers (PUDA, HDDA) and different PUDA ratios during 100k cycles folding test.

| **Cycle number** | **Displacement [μm]** | | | | |
| --- | --- | --- | --- | --- | --- |
|  | **0.1 PUDA** | **0.15 PUDA** | **0.175 PUDA** | **0.2 PUDA** | **0.175 HDDA** |
| **0** | 0 | 0 | 0 | 0 | 0 |
| **1000** | -15.33 | -2.49 | -4.86 | -1.28 | -25.24 |
| **6000** | -8.47 | -15.63 | -1.39 | -0.35 | -20.87 |
| **10000** | -6.51 | -12.18 | -3.44 | -4.06 | -19.49 |
| **20000** | -2.87 | -10.04 | -4.1 | -2.29 | -22.98 |
| **30000** | -4.04 | -6.92 | -0.93 | -3.76 | -29.48 |
| **40000** | -7.26 | -10.04 | -6.47 | -7.58 | -30 |
| **50000** | -14.36 | -8.2 | -4.06 | -5.56 | -32.86 |
| **60000** | -14.65 | -9.2 | -2.97 | -2.47 | -36.37 |
| **70000** | -2.47 | -1.51 | -4.33 | -3.3 | -27.56 |
| **80000** | -9.39 | -5.29 | -5.49 | -8.71 | -27.45 |
| **90000** | -5.7 | -11.7 | -8.79 | -7.84 | -28.86 |
| **100000** | -13.41 | -5.97 | -5.68 | -0.72 | -25.07 |

**Table S10**. The maximum bending stress (*σ_max_*) applied to the OCAs with PUDA and HDDA during folding at a 2 mm radius of curvature.

|  | **0.1 PUDA** | **0.15 PUDA** | **0.175 PUDA** | **0.2 PUDA** | **0.175 HDDA** |
| --- | --- | --- | --- | --- | --- |
| ***σ_max_*** **[kPa]** | 2.00 | 2.40 | 3.23 | 2.60 | 11.40 |

**Table S11.** The displacement changes of OCA (0.175 PUDA) on foldable module during 10k cycles folding test.

| **Cycle number** | **Displacement [μm]** | | |
| --- | --- | --- | --- |
|  | **Top** | **Middle** | **Bottom** |
| **0** | 0 | 0 | 0 |
| **1000** | -0.1 | -2.6 | 0 |
| **2000** | -1.8 | -1.8 | -0.5 |
| **3000** | -1.8 | -3.1 | -1.3 |
| **4000** | -3.3 | -1.9 | -1.6 |
| **5000** | -2.5 | -1.9 | -0.6 |
| **6000** | -1.4 | -1.3 | -0.5 |
| **7000** | -3.8 | -0.6 | -0.3 |
| **8000** | -4.2 | -1.9 | -0.6 |
| **9000** | -2.4 | -0.9 | -1.1 |
| **10000** | -3.5 | -2.6 | -1.6 |

**Table S12.** Stress relaxation ratio of OCAs with different crosslinkers (PUDA, HDDA) and different PUDA ratios.

|  | **0.1 PUDA** | **0.15 PUDA** | **0.175 PUDA** | **0.2 PUDA** | **0.175 HDDA** |
| --- | --- | --- | --- | --- | --- |
| **Stress relaxation ratio [%]** | 58.5 | 54.2 | 41.9 | 37.3 | 64.4 |
